# Non-familial juvenile polyposis of the stomach with gastric cancers: a case report

**DOI:** 10.1186/s40792-018-0488-2

**Published:** 2018-07-24

**Authors:** Tomoko Jogo, Eiji Oki, Minako Fujiwara, Junji Kurashige, Ryota Nakanishi, Masahiko Sugiyama, Yuichiro Nakashima, Hiroshi Saeki, Shinichi Tsuruta, Masataka Nishimura, Yoshinao Oda, Yoshihiko Maehara

**Affiliations:** 1 Department of Surgery and Science, Graduate School of Medical Sciences, Kyushu University, 3-1-1 Maidashi, Higashi-ku, Fukuoka, 812-8582 Japan; 2 Department of Anatomic Pathology, Pathological Sciences, Graduate School of Medical Sciences, Kyushu University, Fukuoka, Japan; 3 Nishimura Internal Medicine and Gastroenterology Hospital, Fukuoka, Japan

**Keywords:** Hamartomatous polyposis, Juvenile polyposis of the stomach, Gastric cancer, Germline mutation, Total gastrectomy

## Abstract

**Background:**

Juvenile polyposis is an autosomal dominant inherited disease characterized by the development of numerous hamartomatous and nonneoplastic polyps of the gastrointestinal tract. Juvenile polyposis has also recently been reported as a predisposition for gastrointestinal cancer.

**Case presentation:**

A 63-year-old man underwent esophagogastroduodenoscopy because of anemia and hypoalbuminemia during a follow-up for gastric polyposis, which showed multiple reddish polyps and two elevated lesions in the stomach. The elevated lesions were diagnosed as well-differentiated adenocarcinomas by biopsy. He had no specific physical findings or family history. Computed tomography showed gastric wall thickening without lymphadenopathy or distant metastasis. Colonoscopy showed an adenoma in the transverse colon. He underwent laparoscopy-assisted total gastrectomy with Roux-en-Y esophagojejunostomy. The resected specimen revealed numerous variously sized non-pedunculated polyps throughout the stomach, diagnosed histopathologically as hamartomatous polyps. The two elevated lesions were diagnosed as a well-differentiated adenocarcinoma restricted to the mucosa and a well-to-poorly differentiated adenocarcinoma invading the submucosa with prominent lymphatic permeation, respectively. Genetic analysis failed to identify any germline mutations in the genes usually associated with juvenile polyposis, including *SMAD4* and *BMPR1A*. However, based on the few characteristic physical findings and histopathological features, the final diagnosis was juvenile polyposis restricted to the stomach.

**Conclusions:**

This patient represented a rare case of non-familial juvenile polyposis of the stomach with gastric cancers. Juvenile polyposis has malignant potential, and patients should therefore be carefully followed up. Surgical treatment, particularly total gastrectomy, is recommended as a standard treatment in patients with juvenile polyposis of the stomach with gastric cancer.

## Background

Juvenile polyposis is a gastrointestinal polyposis characterized by the development of numerous hamartomatous and nonneoplastic polyps [1], with a prevalence of approximately one in 100,000–160,000 [2]. The most frequently affected site is the colorectum (98%), followed by the stomach (14%), jejunum and ileum (6.5%), and duodenum (2.3%) [3]. Juvenile polyposis limited to the stomach is very rare. Some patients have a family history of juvenile polyposis with an autosomal dominant pattern of inheritance, and *SMAD4* and *BMPR1A* have recently been identified as causative genes for juvenile polyposis [4, 5]. Furthermore, although juvenile polyposis is generally recognized as a benign lesion, it has been associated with a predisposition to gastrointestinal cancer in some cases [6–8]. We report a very rare case of a patient with non-familial juvenile polyposis restricted to the stomach and gastric cancers, who was treated by laparoscopy-assisted total gastrectomy.

## Case presentation

**Fig. 1:**
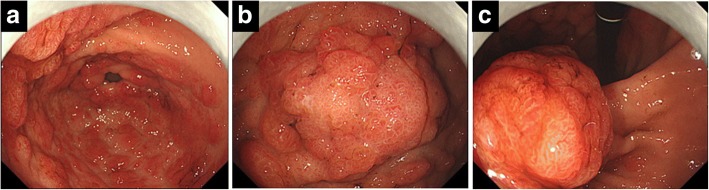
Endoscopic appearance of multiple reddish polyps accompanied by bleeding and erosion throughout the stomach (**a**). Elevated lesions with irregular margins in the anterior wall of the corpus (**b**) and lesser curvature of the angular region (**c**) of the stomach. Biopsy of the elevated lesions revealed them to be well-differentiated adenocarcinomas

**Fig. 2:**
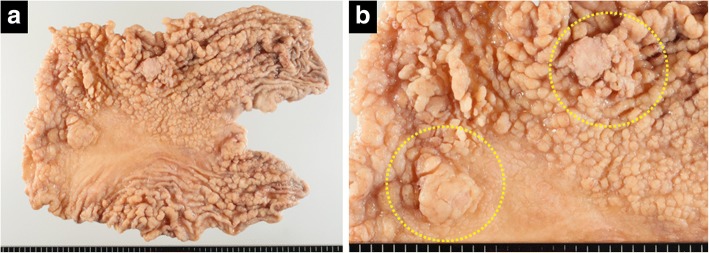
Resected specimen revealed numerous small and large polyps throughout the stomach (**a**) and two elevated lesions in the corpus and angular region (circle), respectively (**b**)

**Fig. 3:**
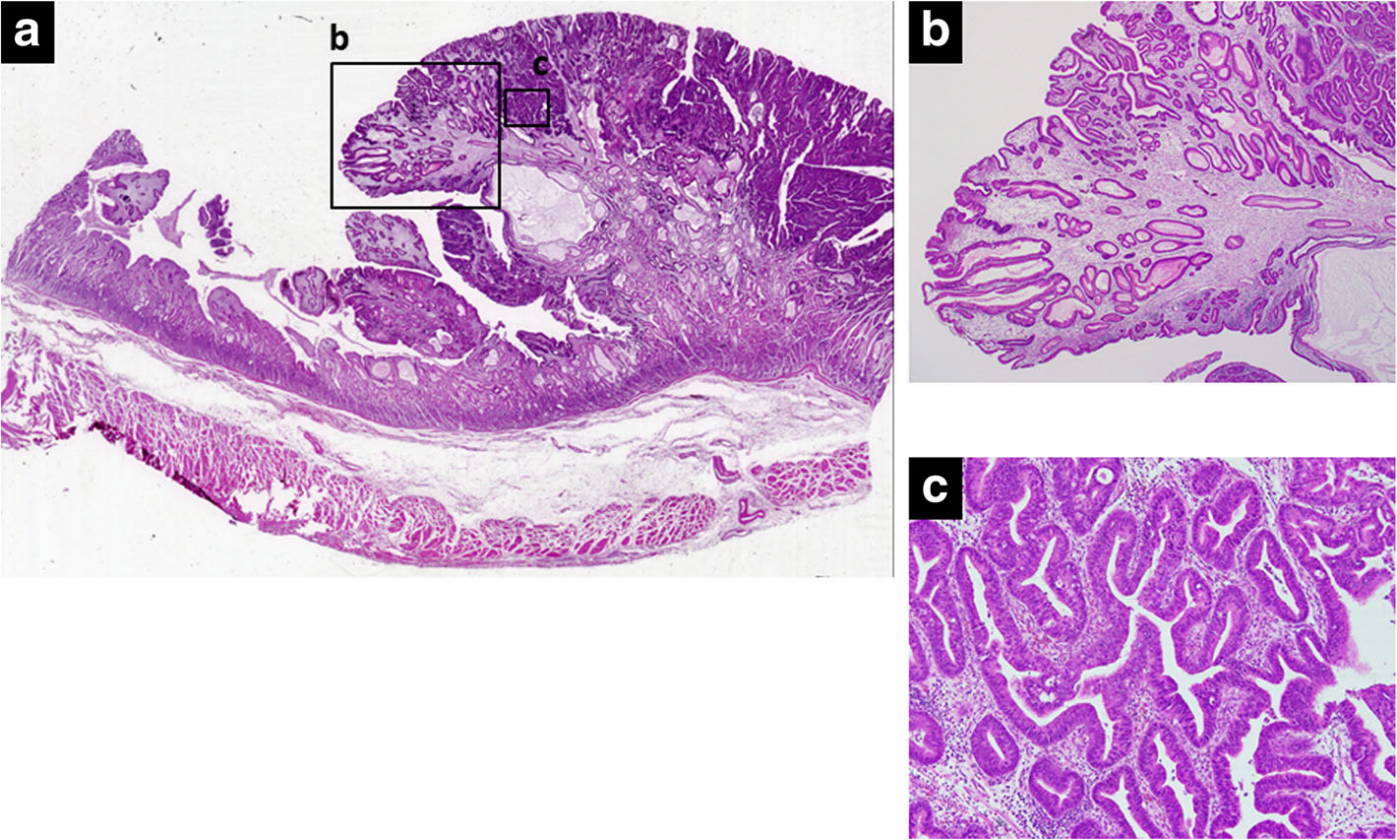
Histopathological examination of the corpus region showed small and large polyps and one elevated lesion (**a**). The polyps comprised hyperplastic foveolar epithelium, cystically dilated glands, and edematous stroma accompanied by chronic inflammation, indicating hamartomatous polyps (**b**). The elevated lesion was diagnosed as a well-differentiated adenocarcinoma restricted to the mucosa, arising in the hamartomatous polyps (**c**). (**a** low-power view, **b** high-power view of square, × 2 objective lens; **c** high-power view of square, × 10 objective lens)

**Fig. 4:**
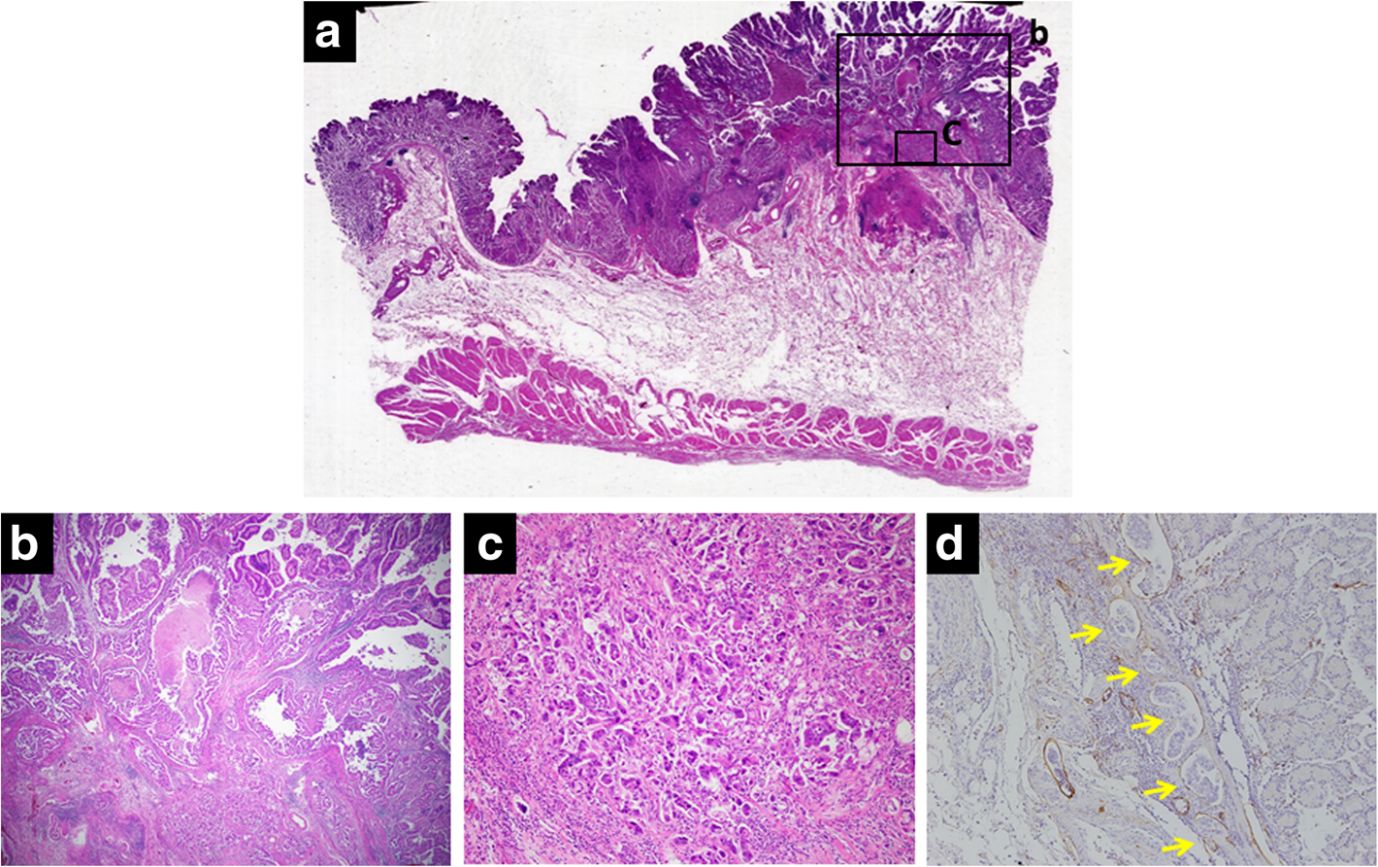
Histopathological examination of the angular region showed the other elevated lesion (**a**). The lesion was a well-differentiated adenocarcinoma in the mucosa (**b**), becoming more poorly differentiated as it invaded the submucosa (**c**). Prominent lymphatic permeation was detected by immunohistochemical staining with D2-40 (arrow) (**d**). (**a** low-power view, **b** high-power view of square, × 2 objective lens; **c** high-power view of square, × 10 objective lens; **d** high-power view, × 10 objective lens)

A 63-year-old man was evaluated for anemia (hemoglobin 11.8 g/dl) and hypoalbuminemia (albumin 3.7 g/dl) in another hospital. He had been diagnosed with gastric polyposis 5 years ago. He underwent esophagogastroduodenoscopy, which showed multiple reddish polyps accompanied by bleeding and erosion throughout the stomach (Fig. 1a) and two elevated lesions with irregular margins in the anterior wall of the corpus (Fig. 1b) and lesser curvature of the angular region (Fig. 1c) of the stomach. Histopathological diagnosis of the two elevated lesions by biopsy showed well-differentiated adenocarcinomas. He was referred to our hospital for treatment of gastric polyposis with gastric cancers. He had no medical history except for gastric polyposis, no family history, and no physical findings such as skin pigmentation or abnormalities of the hair and nails. Blood biochemical tests were negative for tumor markers (carcinoembryonic antigen, 0.6 ng/ml; carbohydrate antigen 19–9, 6.7 U/ml). Computed tomography showed gastric wall thickening, but no lymphadenopathy or distant metastasis. Colonoscopy showed only a polyp in the transverse colon, with a histopathological diagnosis of adenoma. The clinical stage was T1a N0 M0 stage IA according to the Japanese Gastric Cancer Association staging system (14th edition). He underwent laparoscopy-assisted total gastrectomy with D1+ dissection and Roux-en-Y esophagojejunostomy. The resected specimen revealed numerous small and large polyps throughout the stomach and two elevated lesions in the corpus and angular region, respectively (Fig. 2). Histopathological examination showed the polyps to comprise edematous lamina propria with hyperplastic foveolar epithelium and cystically dilated glands, indicating hamartomatous polyps (Fig. 3a, b). The elevated lesion in the corpus was a well-differentiated adenocarcinoma, restricted to the mucosa (Fig. 3a, c). The other elevated lesion in the angular region was a well-to-poorly differentiated adenocarcinoma invading the submucosa (Fig. 4a–c) with lymphatic permeation in the submucosa and muscularis propria detected by immunohistochemical staining with D2-40 (Fig. 4d). The carcinoma showed tubular formation in the mucosa (Fig. 4b), dedifferentiating gradually as it invaded the submucosa (Fig. 4c). Seven of 50 lymph nodes were metastasized by carcinoma cells, which was histopathologically similar to the primary tumor (no. 4d and 7). The final pathological stage was T2 N3 M0 stage IIIA. After receiving informed consent, we analyzed the patient’s genomic DNA to obtain a definitive diagnosis of hamartomatous polyposis. Genomic DNA was extracted from formalin-fixed, paraffin-embedded specimens of hamartomatous polyps and carcinoma, and target genes were comprehensively analyzed by next-generation sequencing with a multiple cancer-associated gene panel. The analysis identified somatic mutations in *APC*, *KRAS*, *TP53*, and *ERBB2* genes in carcinoma, but failed to detect any germline mutations, including in *SMAD4*, *BMPR1A*, or *PTEN*, in hamartomatous polyps and carcinoma. However, based on the few characteristic physical findings and the histopathological features of the polyps, the final diagnosis was juvenile polyposis restricted to the stomach with gastric cancers. The patient was discharged on postoperative day 8 and has been monitored carefully with no adjuvant chemotherapy, by his request. There is no evidence of recurrence 16 months after surgery.

## Discussion

Juvenile polyposis is a rare disease characterized by the development of numerous hamartomatous and nonneoplastic polyps throughout the gastrointestinal tract [1]. Diagnostic criteria of juvenile polyposis include the presence of more than five juvenile polyps in the colorectum, juvenile polyps throughout the gastrointestinal tract, and/or any number of juvenile polyps in a patient with a family history of juvenile polyposis [7]. Juvenile polyposis was reported in 1964 as multiple hamartomatous polyps throughout the intestine, with an autosomal dominant inheritance pattern [9], whereas juvenile polyposis restricted to the stomach was first reported in 1975 [6]. Juvenile polyposis is currently subdivided into three groups according to occurrence site: juvenile polyposis coli, gastric juvenile polyposis, and generalized juvenile polyposis [8, 10].

Hamartomatous polyposis syndrome of the gastrointestinal tract with autosomal dominant inheritance includes not only juvenile polyposis, but also Peutz–Jeghers syndrome and PTEN hamartoma tumor syndrome [11]. In addition, Cronkhite–Canada syndrome is a non-heritable disease that is also characterized by diffuse gastrointestinal polyps, which are histopathologically similar to hamartomatous polyps [12, 13]. Physical findings and family history are helpful for distinguishing between juvenile polyposis and other polyposis diseases. Patients with juvenile polyposis have few physical findings, such as cutaneous hyperpigmentation, dystrophic nail changes, and alopecia, which are detected in patients with Peutz–Jeghers or Cronkhite–Canada syndrome [14, 15]. In addition, patients with juvenile polyposis often show autosomal dominant inheritance, and about 75% of patients have a family history of juvenile polyposis [1]. Recent investigations reported *SMAD4* [4] and *BMPR1A* [5] as genes responsible for juvenile polyposis. Both these genes encode proteins implicated in the transforming growth factor-β signaling pathway [16], which modulates various cellular processes including proliferation, differentiation, migration, adhesion, and death [17]. The prevalence of each of these germline mutations in juvenile polyposis is around 20% [2, 18], while the remaining patients have no such mutations. A confirmed diagnosis of juvenile polyposis is thus based not only on the histopathological features of the polyps, but also on the patient’s physical findings and family history, and on the presence of germline mutations in *SMAD4* and *BMPR1A*. The current patient had no family history of juvenile polyposis or germline mutations in the *SMAD4* and *BMPR1A* genes but was diagnosed with juvenile polyposis of the stomach based on physical findings and histopathological features. Case reports of non-familial juvenile polyposis published in PubMed from 1985 to 2017, including the present case (*n* = 12), are summarized in Table 1 [19–26]. In contrast to the current case, most previously reported patients were relatively young (4–18 years old) and the juvenile polyposis was restricted to the colon and/or rectum, with one case of a single polyp in the stomach [19]. The current case was thus a rare example of non-familial juvenile polyposis.

**Table 1:** Case reports of non-familial juvenile polyposis

Patient no.	Year	Age	Sex	Presentation	Complications	Extent of involvement	Findings	Treatment	Neoplasia	Follow-up after surgery	Result	Reference
1	1986	14	F	Bloody diarrhea, weight loss, facial swelling	–	Colorectum	Multiple polyps	Fulguration, subtotal colectomy	Adenomatous features	24 months	No recurrence	Gilinsky et al. [19]
2	1986	18	M	Bloody stools, lower abdominal cramps, rectal prolapse, weight loss	–	Colon	Multiple polyps	Polypectomy	Adenomatous features	3 years	No recurrence	Gilinsky et al. [19]
3	1986	14	F	Rectal bleeding, abdominal pain, diarrhea	–	Stomach Colorectum	Stomach: a single polyp Colon: multiple polyps	Stomach: polypectomy Colon: polypectomy, fulguration, colectomy	Adenomatous features	24 months	No recurrence	Gilinsky et al. [19]
4	2000	7	F	Diarrhea, abdominal pain, abdominal lump	Intussusception	Ileo-cecum	Multiple polyps	Right hemicolectomy	None	2 years	No recurrence	Panchagnula and Kini [20]
5	2001	15	F	Bloody stools, rectal prolapse	–	Colorectum	Multiple polyps	Proctocolectomy	Adenomatous features	24 months	No recurrence	Bannura et al. [21]
6	2004	4	F	Bloody stools	–	Colorectum	Multiple polyps	Polypectomy	None	2 years	Died	Okada et al. [22]
7	2004	14	M	Abdominal pain, diarrhea	–	Colorectum	Multiple polyps	Proctocolectomy	Adenomatous features	2 years	No recurrence	Chakraborty et al. [23]
8	2007	8	F	Rectal bleeding, syncope	–	Colorectum	Multiple polyps	Total colectomy with rectal mucosectomy	None	6 months	No recurrence	Pratap et al. [24]
9	2007	5	F	Rectal bleeding, rectal prolapse	–	Colorectum	Multiple polyps	Total colectomy with rectal mucosectomy	None	6 months	No recurrence	Pratap et al. [24]
10	2007	17	M	Bloody stools, rectal prolapse	–	Rectum	Multiple polyps	Polypectomy	None	6 months	No recurrence	Tony et al. [25]
11	2017	8	M	Rectal bleeding, abdominal pain, fatigability	–	Colon	Multiple polyps	Total colectomy	None	–	–	Ahmed and Alsaleem [26]
12	2017	63	M	Anemia, hypoalbuminemia	–	Stomach	Multiple polyps	Total gastrectomy	Adenocarcinoma	16 months	No recurrence	Present case

Although juvenile polyposis is a nonneoplastic lesion, several cases have suggested that it represents a predisposition to gastrointestinal cancer [6–8]. The risk of gastric cancer in patients with juvenile polyposis was reported to be approximately 11–21% [27], with a higher risk in patients with a *SMAD4* germline mutation [28]. The current patient had no germline mutations of *SMAD4* or *BMPR1A*, but did have somatic mutations in *APC*, *KRAS*, *TP53*, and *ERBB2* genes. In addition, the histopathological findings showed a well-differentiated adenocarcinoma, or intestinal type according to the Lauren classification [29], directly arising in the hamartomatous polyps in the mucosa. These findings suggest that this case may be classified in the chromosomal instability group among the four molecular subtypes of gastric adenocarcinoma reported by the Cancer Genome Atlas Research Network, because this group was reported to show some salient features such as intestinal histology, *TP53* mutation, and RTK-RAS activation [30].

Accordingly, patients with juvenile polyposis should undergo regular follow-ups, including gastrointestinal endoscopy, with surgical treatment in the event of gastric cancer. However, recurrences of gastric cancer and juvenile polyps in the remnant stomach have frequently been reported in patients who underwent partial gastrectomy [31, 32], and total gastrectomy should thus be considered in patients with juvenile polyposis of the stomach with gastric cancer.

## Conclusions

We report a rare case of a patient with non-familial juvenile polyposis of the stomach with gastric cancers. This case highlights the malignant potential of juvenile polyposis and the need for careful follow-up of such patients. Total gastrectomy is recommended as a standard treatment in patients with juvenile polyposis of the stomach with gastric cancer.
